# A quality improvement study on how a simulation model can help decision making on organization of ICU wards

**DOI:** 10.1186/s12913-024-11161-2

**Published:** 2024-06-05

**Authors:** Danielle Sent, Delanie M. van der Meulen, Andres Alban, Stephen E. Chick, Ilse J.A. Wissink, Alexander P.J. Vlaar, Dave A. Dongelmans

**Affiliations:** 1 Department of Medical Informatics, Amsterdam UMC location University of Amsterdam, Amsterdam, The Netherlands; 2 Jheronimus Academy of Data Science, Tilburg University, Eindhoven University of Technology, ‘s-Hertogenbosch, The Netherlands; 3 Management Department, Frankfurt School of Finance & Management, Frankfurt am Main, Germany; 4 Technology and Operations Management, INSEAD, Fontainebleau, France; 5 Department of Intensive Care Medicine, Amsterdam UMC location University of Amsterdam, Amsterdam, The Netherlands

**Keywords:** Critical care, Computer Simulation, Organizational decision making, Quality of Healthcare, Hospital Bed Capacity

## Abstract

**Background:**

Intensive Care Unit (ICU) capacity management is essential to provide high-quality healthcare for critically ill patients. Yet, consensus on the most favorable ICU design is lacking, especially whether ICUs should deliver dedicated or non-dedicated care. The decision for dedicated or non-dedicated ICU design considers a trade-off in the degree of specialization for individual patient care and efficient use of resources for society. We aim to share insights of a model simulating capacity effects for different ICU designs. Upon request, this simulation model is available for other ICUs.

**Methods:**

A discrete event simulation model was developed and used, to study the hypothetical performance of a large University Hospital ICU on occupancy, rejection, and rescheduling rates for a dedicated and non-dedicated ICU design in four different scenarios. These scenarios either simulate the base-case situation of the local ICU, varying bed capacity levels, potential effects of reduced length of stay for a dedicated design and unexpected increased inflow of unplanned patients.

**Results:**

The simulation model provided insights to foresee effects of capacity choices that should be made. The non-dedicated ICU design outperformed the dedicated ICU design in terms of efficient use of scarce resources.

**Conclusions:**

The choice to use dedicated ICUs does not only affect the clinical outcome, but also rejection- rescheduling and occupancy rates. Our analysis of a large university hospital demonstrates how such a model can support decision making on ICU design, in conjunction with other operation characteristics such as staffing and quality management.

## Introduction

During the 1900s it became clear that clustering of the most critically ill patients was beneficial for their clinical outcomes [1]. As a result of these findings, Intensive Care Units (ICUs) were developed. At present the question has arisen to what degree clustering of ICU patients by their condition is beneficial for patients and society. In other words, whether a specialized ‘dedicated ICU’ is in favor of a ‘non-dedicated ICU’ with a mixed patient population [2, 3]. According to literature, both designs have pros and cons, while strong evidence in favor of one of the two designs is lacking [4–6].

As a result of an ageing population, a continuous increase of healthcare expenditures and shortages in the available medical workforce, a supply-demand mismatch in ICU capacity has arisen in recent years [7, 8]. The capacity strain resulting from this mismatch inevitably leads to increased rescheduling of elective ICU admissions, and to rejection of unplanned patients in need of critical care. To serve as many patients as possible and make efficient use of scarce labor and capital resources, unnecessary capacity loss resulting from unoccupied available beds should be minimized. This raises the question on the ideal ICU design. In this study the non-dedicated ICU refers to general pooling of resources in an ICU with a mixed patient population, and a dedicated ICU refers to an ICU design with specific wards for patients with similar conditions in which dedicated resources are available that are not shared with other wards. In the context of this paper ‘efficient’ is used for a design with as less rejections, rescheduling, and empty beds as possible. In a situation where resources are scarce it is important to be able to see the effects of choices that should be made. By providing a model we give insights into the effects on performance of various designs of ICUs. Based on the principals of pooling, a higher quantity of patients has access to ICU care in a non-dedicated design [9]. Besides, if certain specialisms regularly deal with long-stay patients, this quickly leads to stagnation of admissions and an increase in the number of cancellations in a dedicated design. However, all this only holds when lengths of ICU stays and other process metrics are otherwise the same for both designs.

According to some studies a dedicated-ICU is associated with lower mortality rates, shorter length of stay, shorter duration of intubation and less blood-stream-infections [2, 10–13], whilst other studies were not able to confirm these findings [14–16]. One optimistic paper regarding dedicated ICU was from Mirski et al. which showed a dedicated ICU design to be related to a 25–45% reduced length of stay, resulting in reduced healthcare expenditures [13]. We still find the idea that a dedicated ICU could result in decreased length of ICU stay interesting and want to explore whether these effects could outweigh the efficiency of a non-dedicated ICU. Song et al. as well as Li et al. [17, 18] suggest that behaviours might change in a dedicated ICU, for example in reducing rework or overhead associated with multi-tasking, or with greater sense of ownership, resulting in somewhat shorter length of stay and without loss of quality of care. There is a general trend towards decreased length-of-stay in hospitals has been observed in the past decades [19], and studies show the importance of admissions and discharge policies on ICU performance [18], where admission policy may include features of the likelihood a bed might be immediately available or alternatively a wait or a rerouting of patients might be required, or potentially a need to reschedule elective surgeries. Together, these and other studies suggest that technology and organization changes have roles to play in improving length of stay and quality of care. Taken together, it remains unclear whether a dedicated ICU results in improved outcomes for individual patients. If length of ICU stay and other process metrics do not outperform the efficiency of a non-dedicated design, a dedicated ICU is expected to lead to increased capacity strain which in itself is associated with deterioration in healthcare delivery, suboptimal patient outcomes and decreased job satisfaction among medical staff [7].

In this paper we aim to describe and use a simulation model to help ICU departments to visualize trade-offs between a dedicated and a non-dedicated ICU design. Department-specific characteristics can be imported in such a model to study performance of both designs in terms of bed occupancy, rejection, and rescheduling rates. Depending on local preferences, a model can be optimized for one of the performance measures, at the expense of other measures. In conjunction with other operation characteristics such as staffing and quality management, a simulation model can be useful to gain insights into the effects of decisions that should be made. Besides, models such as these can be utilized to reorganize the ICU department and manage expectations to staff and policy makers in case of abrupt changes in circumstances [20]. We did not also include a wait room, as did [21], because our application was motivated by COVID-19 response, where scenarios of capacity expansion, allocation of that capacity, and the potential need to reroute urgent patients to other facilities, space permitting, was more relevant, as well as the potential role of rescheduling elective procedures in support of urgent critical care needs. That said, the model’s scenario selection allows for an assessment for potential total bed capacity planning, as well as estimates of performance metrics for tradeoffs between allocating that capacity for specialisms versus having a non-dedicated design.

## Methods

This paper followed the SQUIRE 2.0 guideline for reporting on quality improvement studies [22]. The ICU from which admission data are used in this paper, is a large academic hospital in the Netherlands housing a multidisciplinary non-dedicated ICU with a bed capacity of 28 beds divided over four units. INSEAD and Amsterdam UMC cooperated to create a base simulation model which enables to model the performance of a dedicated or non-dedicated design in hypothetical scenarios. It visualizes performance of both designs in terms of rescheduling, rejection, and bed occupancy rates.

Patient inflow data of the local non-dedicated ICU were used, and patients were labeled either as being planned or unplanned. Planned patients were defined as patients that arrived at the ICU via other medical wards in which they had a planned admission. Unplanned patients came straight from the emergency room or from other wards in emergency situations. Arrival, length of ICU stay, rejection, and rescheduling data were also obtained from the local ICU over the years 2015 (*N* = 1881) and 2016 (*N* = 2170). Out of those, 1,779 and 2,043 patients were admitted, and 102 and 127 patients were rejected, respectively. The overall average arriving patients per day was therefore 5.55. In total, 66% of all arrived patients (2,668 patients) were unplanned, and out of them 9% (229 patients) were rejected. Among all patients, 82% (3,117 patients) were admitted on weekdays and 18% (705 patients) were admitted on weekends. Only unplanned patients could be rejected, planned patients were rescheduled if necessary, and in our model the bed partition was, at the expense of rescheduling rates, optimized for rejection rates. Furthermore, all ICU patients were assigned to a medical specialism based on their diagnose on admission (CAPU = cardiopulmonary surgery, CARD = cardiology, INT = internal medicine, CHIR = surgery, NEC = neurosurgery or NEU = neurology or other). The simulation model was coded in R, and an online version is available at https://daniellesent.shinyapps.io/ICU-model/. Parameters of the model were calibrated to hospital scheduling and LOS data for both planned and unplanned patients for each specialism, and bed capacity characteristics of the hospital. Since no association between arriving patients is expected, the arrival of the unplanned patients is assumed to be a Poisson process. The Poisson assumption was tested using the number of unplanned arrivals per day with a chi-squared test and find *p*-values of 0.42 and 0.39, respectively, not rejecting our assumption. The arrival process was estimated independently for weekday and weekend arrivals. The arrival of planned patients is follows a categorical distribution, where the probabilities are given by the fraction of days in the two-year period that a given number of patients arrived. The distribution family of the LOS was chosen using the tool Stat::FitTM. The simulation ran for a period of 3,770 days (approximately ten and a half years), where the first 120 days were burned-in to warm up the queue. The remaining ten years were use for evaluation purposes. The model was verified by checking several special cases of inputs to theoretical values provided by queueing theory. More in depth specifications of the model and it evaluation can be found in the article of Alban et al. [23].

For this study, we extended the base model to be able to run four clinically relevant scenarios that help decision-making on ICU design under varying hypothetical circumstances. First the base case model will be shown with a maximum capacity of 28 beds, the total number of beds that were available at the ICU of the hospital the data was obtained from. The base case model compares a dedicated with a non-dedicated ICU design. In this scenario the dedicated ICU is divided into four specialized units, based on the current infrastructure of the building. This structure uses a partition of six beds for unit CAPU, eleven beds for a combination unit of CARD/INT/Other, five beds for unit CHIR and six beds for a combination unit of NEC and NEU. This partition was found to be optimal for 28 beds in terms of all three performance measures. Yet, in practice bed capacity fluctuates due to specific circumstances such as workforce constraints, a pandemic or holidays either increasing or decreasing the number of beds available for patients. Therefore, a second model shows the performance for a dedicated and a non-dedicated ICU under varying bed capacities, to complement other studies of ICU dedicated versus flexible capacity [17], which may be influenced as well by patient mix [21]. A third scenario visualizes the situation in which a dedicated ICU design decreases length of ICU stay (LOS) in advance of a non-dedicated ICU, as was shown by Mirski et al. [13]. Finally, a scenario with an increased inflow of unplanned patients is simulated, motivated by the COVID pandemic in which the inflow increased dramatically. To do so, a new simulation population was created by decreasing the original inter-arrival rates. Taken together, the graphs may show the numbers of beds required in dedicated or non-dedicated settings in order to achieve a given threshold for metrics of interest, thereby presenting a guage with which to assess potential behavioural effects that might be active [18]. Ethical approval was not required, only anonymized data containing date and time of admission and (if applicable) departure and specialism were used for the model. No further patient data was used.

## Results

**Fig. 1:**
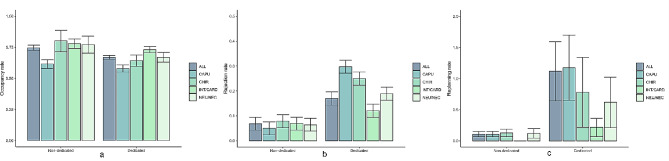
Comparison of the non-dedicated and dedicated ICU in terms of occupancy (**a**), rejection (**b**) and rescheduling (**c**) rates in base-case situation

The simulation of the first scenario is shown in Fig. 1 and presents the base-case model with 28 beds for a non-dedicated and a dedicated ICU design in terms of a (occupancy), b (rejection) and c (rescheduling) rates.

Each measure is reported for the overall performance and for performance of the four different clinical diagnose groups (CAPU, CHIR, INT/CAR, NEU/NEC). As the general ICU is not divided into units, the values correspond to the performance specific to the cluster of patients assigned to the respective dedicated unit. The overall rejection rate for the non-dedicated ICU is 7.7% versus 18.1% for the dedicated ICU. The overall rescheduling rates are 13.0% versus 100.5% (meaning that some patients had to be rescheduled more than once). The overall occupancy rate lies lower in the dedicated ICU (75.1% versus 67.4% for non-dedicated and dedicated respectively). Note that the non-dedicated ICU has lower rejection and rescheduling rates despite the higher occupancy rate. The rescheduling simulation model shows a high standard error, which originates from the fact that the number of rescheduled patients per day is mostly very low, and binary (yes rescheduled or not rescheduled).

**Fig. 2:**
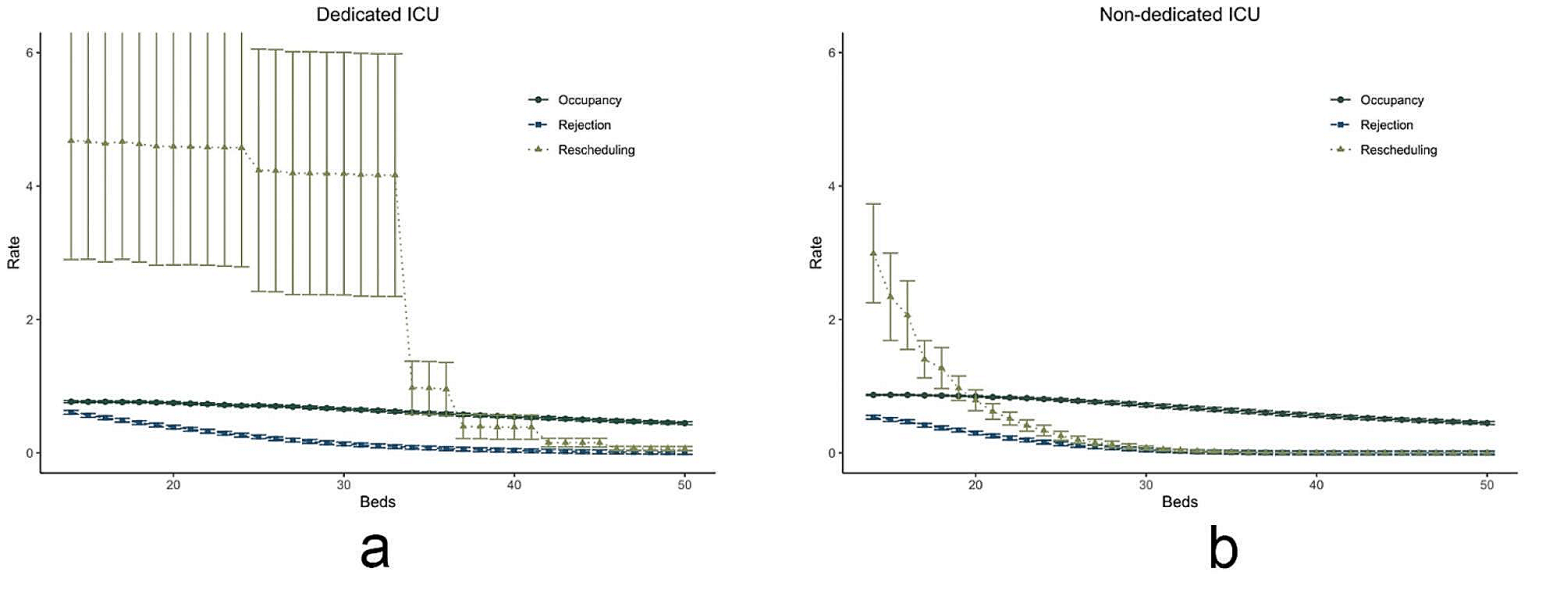
Performance of a dedicated (**a**) and a non-dedicated (**b**) ICU with a varying bed capacity

Figure 2 shows the performance of a dedicated (a) and a non-dedicated (b) ICU with varying bed capacity, ranging from 14 to 50 beds. While the non-dedicated ICU starts out with a higher occupancy rate than the dedicated ICU (86.9% vs. 76.8%), both end up with a similar occupancy rate at the capacity of 50 beds (44.9% vs. 44.6%). Between the minimum and maximum bed capacity that we tested, the occupancy rate in the non-dedicated ICU is constantly higher than in the dedicated ICU. Note that for the dedicated ICU it is observed that at a capacity of 34, 37, 42 and 46 beds, the rescheduling rate drops. At these steps, the capacity for the specialism with most planned patients was increased with one bed, resulting in dropping of the rescheduling rate. For the non-dedicated ICU, the rejection rate and rescheduling rate both come close to their minimum around a bed capacity of 30 beds, while for the dedicated ICU this is seen at a bed capacity of 45 beds. The rescheduling rate is steadily higher than the rejection rate. The comparison of Fig. 2a and b also allows for comparison of the minimum number of beds required to reach a rejection rate below 5%, which is a target in ICU organization management in the Netherlands [24]. In the non-dedicated ICU setting, 30 beds are required to meet this target, while in the dedicated ICU 38 beds would be needed. With a bed capacity of 30 beds, the non-dedicated ICU would have an average occupancy of 71.7%, while a bed capacity of 38 beds in the dedicated ICU leads to an average occupancy of 56.3%. The average rescheduling rates for this number of beds lie at 7.4% - and 39.4%, for the non-dedicated and dedicated ICU respectively.

**Fig. 3:**
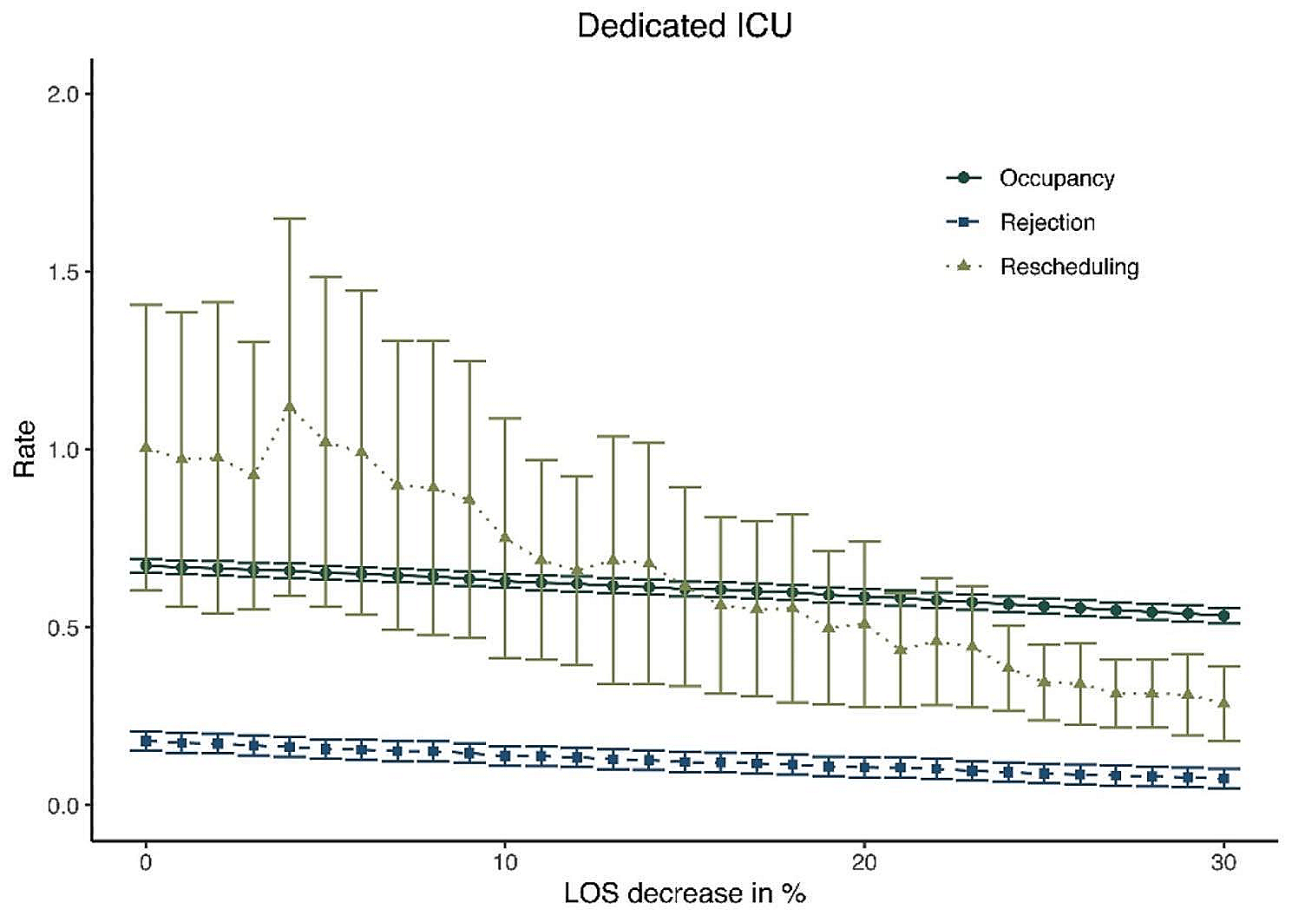
Hypothetical lengths of stay reduction in a dedicated ICU design

Figure 3 shows a simulation of the dedicated ICU setting, where an average length of ICU stay (LOS) reduction is assumed. It demonstrates that in the dedicated ICU, bed occupancy rates decrease steadily as the average LOS becomes shorter. In the base-case situation, with no LOS reduction, the overall occupancy rate was 67.4% in the dedicated ICU, while at a hypothetical 30% LOS reduction it lies at 53.3%. With a hypothetical 30% reduction in LOS, rejection rates decrease to 7.5% and rescheduling rates decrease to 28.6%. As described in the first scenario, in the non-dedicated ICU setting the average rejection, rescheduling and occupancy rates were 7.7%, 13% and 75.1%, respectively.

**Fig. 4:**
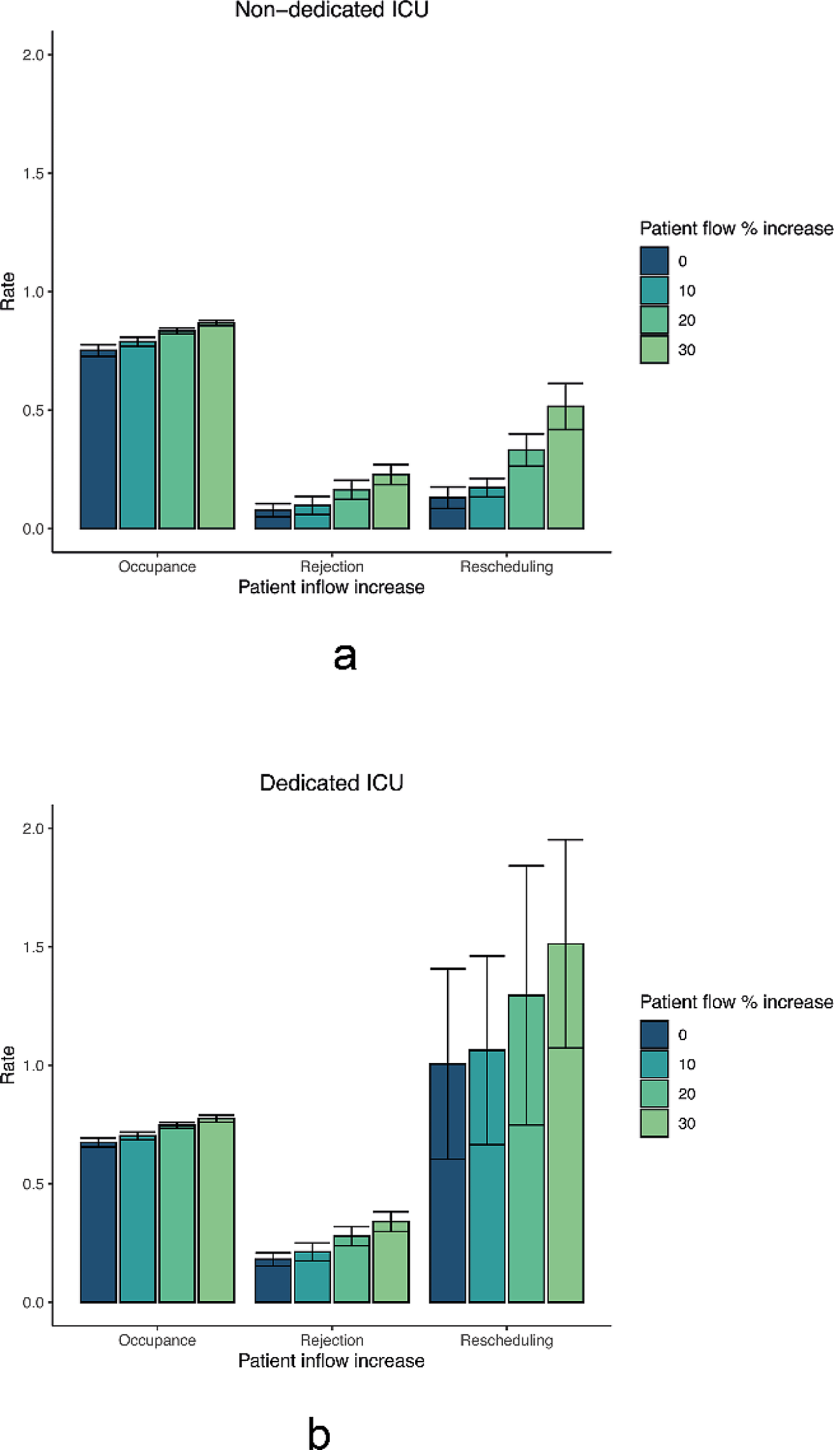
Increase in unplanned patient inflow in a non-dedicated (**a**) and a dedicated (**b**) ICU design

The last scenario is shown in Fig. 4 for a non-dedicated design (a) and a dedicated design (b). In this scenario the simulation model ran for various increases (10%, 20% and 30%) of unplanned patients of each specialism. The 0% increase bar shows the results for the base-case patient inflow. In the non-dedicated ICU design the occupancy rates are 75.1%, 78.8% and 83.4% for a patient inflow of 0%, and an increase of unplanned patients of 10%, 20% and 30% respectively. In the dedicated ICU the occupancy remains lower with rates of 67.4%, 70.2%, 74.7% and 77.5% respectively. In the non-dedicated ICU setting the rejection rates (7.7%, 9.7%, 16.3% and 22.8%) and rescheduling rates (13%, 17.2%, 33.1% and 51.5%) increase gradually as the inflow of unplanned patients increases. In a dedicated ICU design the rejection rates are higher for the same increase in inflow, 18.1%, 21.3%, 27.9% and 34.0%, respectively. The rescheduling rates for 0, 10, 20 and 30% increase in unplanned patient inflow are 100.5%, 106.3%, 129.5% and 151.2% respectively, meaning that some patients will be rescheduled more than once.

## Discussion

This study aimed to provide insights in the capacity performance of a dedicated ICU in comparison with a non-dedicated ICU by using a discrete event simulation model. Previous literature shows no consensus on a preferred design [2, 10, 12–16]. However, it is known that organising a non-dedicated ICU has its challenges because effective management is amongst other factors depending on the establishment of continuous professional development, ensuring that all personnel is equipped to respond to the diverse needs characteristic of non-dedicated ICUs we note that our simulations do not answer the question of whether a non-dedicated ICU should or should not be preferred over a dedicated ICU. We quantify the performance, in terms of the number of rejected and rescheduled patients and the occupancy rate of the ICU’s. Our simulation model was developed using admission data of a large university hospital ICU in the Netherlands.

The first scenario showed that the non-dedicated ICU design dominates the dedicated ICU design in terms of occupancy, rejection, and rescheduling rates in the base-case scenario. The second scenario showed that the dedicated ICU needed eight more beds to accomplish a desired rejection rate below 5%, when compared to the non-dedicated ICU design. Yet, the model showed that scaling up with eight beds to a total of 38 beds in a dedicated design resulted in a bed occupancy rate of 56.3%, which can be seen as inefficient use of expensive and scarce ICU resources. The non-dedicated ICU should scale up with at least two more beds to be able to have a rejection rate below 5%, as is described in the national guidelines [24]. Figure 2b shows that the rescheduling rates for the dedicated ICU remain high, up to a high number of available beds. This is likely because in our analysis the bed partition was optimized for rejection rates, clearly at the expense of rescheduling rates. The third scenario explored the theory that a dedicated ICU leads to a shorter average LOS due to the specialization of healthcare delivery as was shown by Mirski et al. in 2001 [13]. The simulation model showed that if a dedicated design would lead to a 30% reduction in average LOS, the non-dedicated ICU would still outperform the dedicated ICU in terms of lower rescheduling rates and higher occupancy rates of available beds. The rejection rates for the dedicated (7.5%) and non-dedicated (7.7%) ICU would be almost equal. One might wonder whether a significant decrease in LOS is still realistic today. The fourth scenario studied unexpected increases in unplanned patient inflow. The non-dedicated ICU adapts easier to the situation in terms of higher bed occupancy rates. In the case of a 30% increase in unplanned patients, 22.5% of the beds remains empty in a dedicated design, while this is 16.6% for the non-dedicated ICU. Thus, more beds remain empty while at the same time rejection and rescheduling rates increase in the dedicated ICU.

The simulation models made clear that from a resource efficient perspective a non-dedicated ICU outperforms a dedicated ICU. Yet, some studies found improved individual patient outcomes in a dedicated ICU design [2, 10–13], whilst others did not [14–16]. We quantified some surprising ‘jump’ effects for the specialisms that some other papers have not seen – e.g. when there is a long-hauler. Song et al. [17] as well as Li et al. [18] suggest that behaviours might change in the dedicated – a greater sense of ownership of patients, and in our case greater sense of specialization for a given specialism. This would suggest that using the same service rates for both the simulation of the non-dedicated ICU as well as the dedicated ICU, might not be realistic, since dedicated ICU’s might have slightly better length of stay for instance. In this light, it should be noted that it remains unclear whether these potential positive effects in the dedicated ICU are associated with the level of specialization or are a result of structural lower bed occupancy rates in this setting. Contrary to low bed occupancy, high occupancy rates could result in higher rejection and rescheduling rates, which are associated with higher mortality rates and inferior patient prognosis [7, 25–29]. Hence, the previously found positive effects of a dedicated design in some studies could also have been effects of structural lower bed occupancy rates in this setting, instead of being an effect of a higher level of specialization. Furthermore, specialization and impact on outcome may also follow a U-shape curve in which higher level of specialization may also result in not being able to diagnose and treat complications of another domain. Taken together with other studies that did not find evidence for improved outcomes for individual patients in a dedicated ICU design, the potential gains of a dedicated design remain uncertain, while capacity benefits of a non-dedicated design are evident [14–16].

Strengths of this study are the development and application of a simulation model that quantifies trade-offs that are important in capacity management in ICUs. ICU’s can use local admission data to personalize the model. Limitations of this study are that while designing our simulation model, we were not able to include daily practice issues such as the limitation of the number of available beds due to sickness or holiday leave of nursing staff, changes in inflow of patients throughout the year, etcetera. The simulation furthermore quantifies three capacity trade-off measures, but does not include assessment of quality, survival, service times, costs and other measures that are also involved in the multi-criteria decision of ICU design. Further, the simulation model was only used in one single-center case, and we did not explore other ICU designs or more flexible models. Other industries showed for example that if products are interchangeable in a multi-factory supply network (long-chain-model), in which each factory has a backup (e.g. factory one has two as backup, factory two has three as backup, etc.) then the performance improves [30]. However, for ICU care delivery, it is unknown what the quality and safety effects are if for example a dedicated surgery patient, is admitted to a dedicated cardiology ward.

Future research could expand the simulation model and add for example a dedicated ‘isolation’ unit to support ICU design decision making during a pandemic such as COVID-19 or outbreak of more regular infectious diseases that require patients to be treated isolated from other patients.

## Conclusion

The model we present shows how a simulation can be utilized to make trade-offs between clinical goals in terms of rejection and rescheduling rates and efficiency in terms of occupancy rates. It helps to find a bed occupancy rate at which the rejection and rescheduling rates are acceptable for a specific ICU in different scenarios. The insights gained from the model can support decision making on the local ICU design. The model showed that a non-dedicated design outperforms a dedicated ICU in terms of higher efficiency. These metrics, as a function of capacity and design, are useful inputs to complement local data on quality and local specialization skills to support a local ICU design decision.

## Data Availability

The datasets used and/or analysed during the current study are available from the corresponding author on reasonable request. Online access to the R code of the event simulation model is available on reasonable request.
